# Systematisation of spatial uncertainties for comparison between a MR and a CT-based radiotherapy workflow for prostate treatments

**DOI:** 10.1186/1748-717X-4-54

**Published:** 2009-11-17

**Authors:** Tufve Nyholm, Morgan Nyberg, Magnus G Karlsson, Mikael Karlsson

**Affiliations:** 1 Department of radiation sciences (Oncology), Umeå University Hospital, 90187 Umeå, Sweden; 2 Information and Communication Technology, Luleå University of Technology, Sweden; 3 Department of radiation physics, Umeå University Hospital, 90185 Umeå, Sweden; 4 Radiation physics section, Department of radiation sciences, Umeå University, 90187 Umeå, Sweden

## Abstract

**Background:**

In the present work we compared the spatial uncertainties associated with a MR-based workflow for external radiotherapy of prostate cancer to a standard CT-based workflow. The MR-based workflow relies on target definition and patient positioning based on MR imaging. A solution for patient transport between the MR scanner and the treatment units has been developed. For the CT-based workflow, the target is defined on a MR series but then transferred to a CT study through image registration before treatment planning, and a patient positioning using portal imaging and fiducial markers.

**Methods:**

An "open bore" 1.5T MRI scanner, Siemens Espree, has been installed in the radiotherapy department in near proximity to a treatment unit to enable patient transport between the two installations, and hence use the MRI for patient positioning. The spatial uncertainty caused by the transport was added to the uncertainty originating from the target definition process, estimated through a review of the scientific literature. The uncertainty in the CT-based workflow was estimated through a literature review.

**Results:**

The systematic uncertainties, affecting all treatment fractions, are reduced from 3-4 mm (1Sd) with a CT based workflow to 2-3 mm with a MR based workflow. The main contributing factor to this improvement is the exclusion of registration between MR and CT in the planning phase of the treatment.

**Conclusion:**

Treatment planning directly on MR images reduce the spatial uncertainty for prostate treatments.

## Background

**Figure 1:**
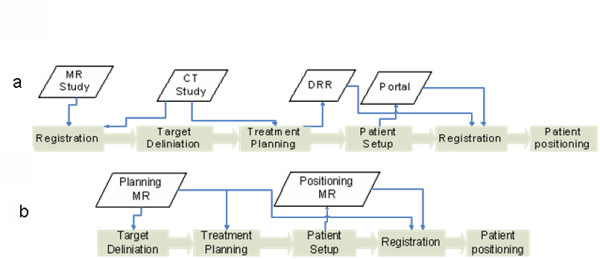
**Overview of the two workflows analyzed in the present study**. (a) A widely used workflow utilizing registration between MR and CT images in order to transfer the delineated prostate volume (GTV/CTV) from the MR study to the CT study. The CT study is used for treatment planning and to generate DRR's for patient position. Typically, fiducial markers are used. (b) The workflow is entirely based on MR, both for planning and positioning.

MR images are well suited for target delineation, not only for the prostate [1], but also for many other tumours, such as brain lesions [2,3] and head and neck tumours [4,5], which explains the growing interest for MR in radiotherapy [6-12]. An "open bore" 1.5T MRI, has been installed in direct connection to a treatment unit at the radiotherapy department in Umeå [13]. This installation allows us to image most of our patients in treatment position with the MR for the target delineation, and open the door for development of an online treatment setup workflow designed for soft tissue tumours. Figure 1 illustrates a MR-only workflow and a more conventional CT-based workflow. In the MR-based workflow, the target definition, the treatment planning, and patient positioning at treatment delivery, are performed with MR aid only. The patient positioning utilize a transport trolley to move the patient from the imaging in the MR to the treatment table. A very robust fixation of the patient provides control over the relation among the coordinate systems in the patient, in the MR, and in the treatment room. However, the transport does introduce uncertainties, which must be accounted for in an evaluation of the workflow and the resulting geometric uncertainties.

An alternative workflow could be to plan on MR material followed by positioning based on fiducial markers. This intermediate workflow requires that the internal markers are visible on the MR images and that the apparent marker positions are correct. Parker et al. [14] shows that internal markers appear clearly on gradient echo sequences, while more difficulty to identify on T2-weighted turbo spin echo sequences. The visibility of the markers was increased when the TE time was reduced, giving higher signal but compromising the T2-weighted contrast. Verified robust imaging of fiducial markers in MR would enable also this workflow. In the present study, this intermediate workflow will not be explicitly handled.

The purpose of this study is to investigate if a MR-only radiotherapy workflow, in accordance with figure 1b, has the potential to improve the spatial accuracy compared to the more conventional CT-based workflow (figure 1a). The estimations of the uncertainties in the different workflows are based on both a literature review and the results of our own experiments.

## Methods

In order to assess the total spatial uncertainties in the two workflows, shown in figure 1, the workflow processes were broken down into independent sub processes. Both workflows contain two main steps where uncertainties can be introduced, target definition for treatment planning and patient positioning at treatment delivery. Our tools in the uncertainty analysis have been literature reviews, and when necessary own experiments. The own experiments concern positioning with MRI, and are described in the section about MR guided delivery.

An open-bore MRI scanner (Siemens Espree, 1.5T) was used for the MR imaging of the patients in connection radiotherapy. For prostate patients, a T2-weighted SPACE sequence (Siemens), which is a 3D turbo spin-echo sequence with varying flip angle on the refocusing pulses, was used. The slice thickness was 1.7 mm, typical pixel-size was 1.0 × 1.0 mm^2^, and the bandwidth was 592 Hz per pixel. Distortions caused by gradient non-linearity were corrected with an algorithm based on spherical harmonic expansion of the fields generated by the gradient coils [15]. The 3D correction algorithm including representation of the coils was delivered by Siemens as a standard clinical tool integrated in the scanner software (VB15). The scanner was set in an isocentric mode, which moves the table prior to the acquisition of each sequence, to place the MR isocenter in the centre of the volume of interest.

The total spatial uncertainty consists of both a random part, varying in direction and magnitude from fraction to fraction, and a systematic part, which is invariant over the treatment period. The systematic and random uncertainty should be given different weight in the formation of margins between the CTV and the PTV. In the present work we used the weight factor 2.5 for the systematic errors and 0.7 for random errors as proposed by van Herk et. al. [16,17]. The PTV margin is hence expressed as(1)

where Σ is the systematic and *σ *is the random spatial uncertainty. The presented uncertainties are throughout this paper presented in units of one standard deviation (1SD), thus inherently assuming normal distributed data.

### Uncertainty in target definition

The total uncertainty in the target definition can be broken down to three subparts: uncertainty in prostate delineation (MR-based on both workflows), spatial distortion in MR images that can be scanner related and patient induced, and for the CT-based workflow: uncertainty in registration between CT and MR images.

#### Uncertainty in prostate delineation

Rasch et al [18] has from a study with 18 patient analysed by 3 physicians reported an uncertainty, in the prostate delineation on axial MR study, of 2 mm at the base of seminal vesicles and up 2.8 mm in the prostate apex. The uncertainty in the head-feat (HF) direction was 2.5 mm with a slice thickness of 5-6 mm for the axial MR images. In a later study involving 7 physicians analysing 10 patients Smith et al. [19] reported a radial uncertainty of 0.6 - 1.6 mm for the delineation of the prostate where the larger value is for the apex. The inter-observer uncertainty in the length (HF direction) of the prostate was 3.4 mm, and the intra-observer variation was 2.6 mm; the slice thickness was 2.5 mm.

In summary, the literature review indicates a prostate delineation uncertainty of 1.8 mm in the right-left (RL) and anterior-posterior (AP) directions and 2.8 mm in the HF direction.

#### Geometrical Distortions in MR

Geometrical distortions in MR images are a well known phenomena [20-22]. In modern MR scanners, gradient non-linearity is the main cause of image distortions [20], dominating over the effect of static field inhomogenity. The distortions introduced by the gradient non-linearities are increasing with the distance from the MR isocenter [20,23] Without correction, the geometrical distortions in modern MR scanners can cause deviations between physical and imaged distances of up to 20% in extreme situations. However, there are methods for distortion correction which reduces the errors significantly. It is possible to use a specially designed geometry phantom to characterize and correct the distortions for a specific scanner [22,24]. In the present work, a gradient coil specific distortion correction algorithm was applied. Even though this device specific corrections only correct for intrinsic gradient non-linearity connected to a specific type of scanner/gradient coil, it has been shown that this kind of correction yields a spatial accuracy better than 2% [23,25], which is sufficient when region of interest in the patient is close to the MR isocenter. Patient anatomy, e.g. air pockets in the rectal cavity, can generate susceptibility-generated field changes up to ± 10 ppm [26]. With a bandwidth of 592 Hz per pixel this corresponds to distortions up to approximately 1 pixel for a 1.5T scanner. Thus, magnetic susceptibility related distortions are a minor effect for the sequence used.

In summary, for a prostate with radius of 2.5 cm the geometrical distortions can cause errors of up to 0.5 mm, which corresponds to a standard deviation of around 0.2 mm. This uncertainty is approximately equal in all directions provided that a 3D correction algorithm is used.

#### Registration uncertainties - MR/CT

The workflow in figure 1a involves a registration between a CT and MR study. Errors in this registration directly affect the spatial accuracy of the target definition. Registrations between MR and CT for prostate patients can be performed based on fiducial markers [14]. The trend is, however, to use mutual information (MI) registration based directly on the patient anatomy [27,28]. The prostate position relative other anatomical structures is not fix, therefore the registration should ideally be based on the prostate with just a small margin. However, this has been reported problematic because of too limited morphological information content in the CT representation of the prostate [29,30]. A few studies have been performed evaluating the accuracy and precision of MI registration for CT and MR studies of the prostate; the registration uncertainty has been reported to be around 2 mm [29,31]. Roberson et al. [31] reported that registration results depend on the starting point for a specific MI optimization software. The mean difference between different stating points was up to 1 mm in the RL direction. The corresponding number for MR-MR registration was 0.4 mm in the HF direction which could indicate that the mutual information maximum is more distinct for MR-MR registration compared to CT-MR registration.

In summary, the registration uncertainty for a CT - MR registration for a prostate case was estimated to be 2 mm based on current reports in the scientific literature.

### Uncertainty in patient positioning

The patient positioning at treatment, with the development of image guided radiotherapy, been in focus the recent years. For prostate cancer patients the improvements in spatial treatment accuracy has been considerable. Both the CT and the MR-based workflows, shown in figure 1, rely on imaging before each fraction. Intra-fraction motion of the prostate is therefore an issue for both workflows.

#### Intra-fraction prostate motion

In a large investigation by Kotte et al [32] intra fraction motion larger than 2 mm was observed during 66% of the fractions, this number is roughly in agreement with the results presented in other studies [33,34]. However, reduction of the rectal filling has been showed to be of great importance to achieve a stable prostate position [33,35]; an uncertainty of 2 mm is therefore realistic for a 5-7 min treatment when patients are instructed to empty rectum prior to treatment. The position uncertainty due to prostate motion is most pronounced in the AP and HF directions [32,36].

In summary, the overall uncertainty for the prostate position was estimated to 2 mm, which broken down in the orthogonal directions corresponds to: 1.4 mm in AP and HF, and 0.4 mm in RL.

#### Uncertainty with fiducial markers

There are numerous studies on the accuracy of patient positioning using fiducial markers and portal or flat screen kV images. Several different sources of uncertainty need to be considered in order to correctly estimate the overall accuracy of the workflow. Random positioning errors are partly due to uncertainty in the registration between the reference image and the portal/kV flat screen image. Literature indicates that a manual registration typically results in uncertainty of around 0.7 mm in the HF and RL direction, and 1.4 mm in the AP direction [37,38].

An investigation by Nichol et al [39] indicates that a systematic deformation of the prostate during radiotherapy leads to drift in the relation between the centre of mass for the markers and centre of mass for the contoured prostate. This uncertainty is in the order of 1 mm, which is roughly in agreement with other reports [40,41]. It should be noted that deformation of the prostate is in many respects equivalent to marker migration within the prostate. These two effects are therefore not separated in the present work. Prostate deformation and marker migration are resulting in a systematic uncertainty in the patient position.

The uncertainty of clinical imaging systems are in the order of 1 mm, accounting for limitations in resolution, isocenter position and mechanical instability. Paulsen et al [34] observed a systematic discrepancy of almost 1 mm when comparing 2 different imaging modalities at 2 different accelerators. Kotte et al. [32] detected that the sag of the gantry caused a systematic imaging deviations of almost 1 mm in the HF direction when the gantry was in 0 degree position compared to 180 degree position.

In summary, it is estimated that the uncertainty in the day to day registrations between reference image and the portal image is 0.7 mm in RL and HF direction and 1.4 mm in AP direction. The estimated uncertainty for the marker position in the prostate is 1 mm in all directions, and the estimated total uncertainty for the imaging systems is 1 mm in all directions.

### MR guided treatment delivery

The MR positioning approach is novel; we therefore describe the principals in detail below, as well as the experiments performed to estimate the uncertainties connected to the method.

**Figure 2:**
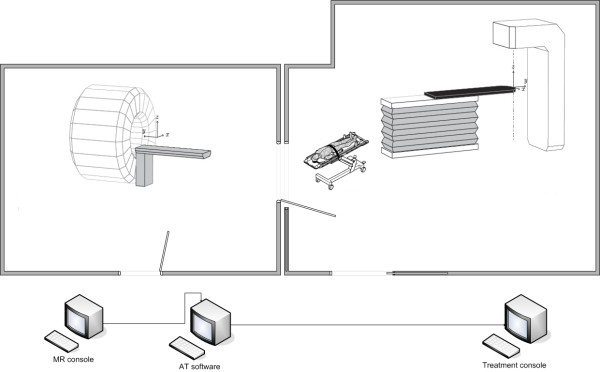
**Schematic overview of the hardware configuration for the MR positioning of patients**. There is a direct connection between the MR room and treatment room, which makes patient transport quick and simple. In parallel with the patient transport the treatment couch coordinates are calculated using dedicated image registration software, the transport in it self does therefore not prolong the procedure.

Figure 2 shows the hardware configuration. The patient is transported between the MR scanner and the treatment unit on a MR compatible trolley (Miyabi, TRUMPF). The patient is fixated on a shell, with a double vacuum system (BodyFIX, Medical Intelligence an Elekta company), which can be slid from the trolley to the treatment or MR table after docking. The shell has fixed positions both at the MR and the treatment table, which enables absolute coordinate transformation between MR coordinates and treatment coordinates. The treatment table is a Siemens 550 TxT equipped with a modified TT-D table-top compatible with the Miyabi transport solution. The daily treatment table coordinates are calculated as the absolute table coordinates from the treatment planning corrected for daily variations in patient and prostate position. The daily correction is calculated based on a sub-volume-based rigid mutual information registration between the reference MR images used at treatment planning and daily positioning MR images. The same SPACE sequence was used both for treatment planning and for daily positioning. Calibration of the system, i.e. determination of the absolute coordinate transformation vector, is an obvious source for systematic uncertainty, while mechanical instabilities in the mounting mechanism at the MR and treatment table together with image distortion, image registration errors and patient movement during transport mainly result in uncertainty of random nature.

#### Uncertainty in calibration vector determination

**Figure 3:**
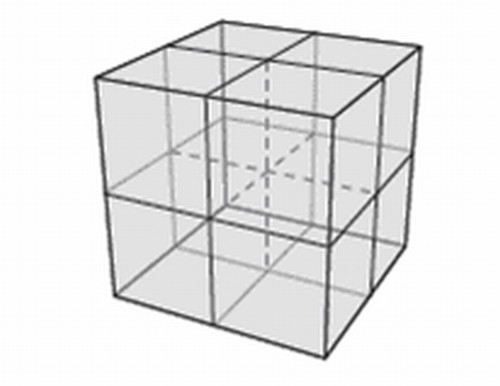
**Calibration phantom**. The phantom which was used for coordinate calibration is 15 × 15 × 15 cm3 and filled with water. The central point is defined with lead bullet of 1 mm diameter which is fasten with 6 thin plexi rods creating a 3D hair cross.

The calibration vector is the relation between the coordinate for a specific point, in the MR coordinate system and the treatment table coordinates that brings the same point to the treatment isocenter. The calibration vector was determined using a phantom which is sketched in figure 3. The centre point of the phantom is clearly visible on MR, CT, portal images and can also be positioned using lasers. We placed the phantom at various positions on the Miyabi shell and carefully determined the position of the centre point in both the MR coordinate system using MR images, and the treatment coordinate system using calibrated lasers. The calibration vector was calculated, for each phantom position on the Miyabi shell, as the difference between the MR coordinates and the treatment table coordinates for the central point in the phantom. The idea with repeated measurements was to assess the precision of the vector determination taking intrinsic inhomogeneities in the magnetic field and position dependent distortions into account. In total 16 independent determinations of the calibration vector was performed, for different phantom positions on the Miyabi shell. The measurements were performed with the phantom centre positioned at ± 25 mm in the AP direction, and ± 60 mm in the RL direction and at 4 different positions along the HF direction with a total span of 450 mm. The scanning of the phantom was performed in isocentric mode.

#### Weight correction

The calibration vector needs to be corrected based on the patient's weight to account for the treatment table sagging. The magnitude of the sagging was investigated using a set of 15 kg bricks which were distributed to approximate the weight distribution of a typical patient. We varied the total load and the weight distribution on the table top, to simulate patient weight from 0 to 105 kg, and patient height from approximately 150 cm to 190 cm.

#### Geometrical distortions

The prostate is typically located on the patient's central line and with the Miyabi shell together with the BodyFIX vacuum pillow the height of the prostate for the typical patient will be very close to the isocenter. The internal MR laser is used to position the patient in the HF direction before imaging, thus the prostate will be close to the isocenter also in the HF direction. If the prostate centre is within a sphere of 5 cm around the MR isocenter and the maximum spatial distortion is 2% then the maximum error will be approximately 1 mm, i.e. a standard deviation around 0.5 mm. The geometrical distortions systematically affect the entire treatment through the reference images, and do in addition contribute to random errors at each fraction.

#### Patient movement

Significant patient movements during the time interval from the imaging to the treatment are deemed highly unlikely when using the double vacuum immobilization device. There is however a risk for prostate movements within the body during this time interval as discussed above (see section about intra-fraction prostate movement)

#### Position reproducibility

The reproducibility of the Miyabi shell position on the MR and treatment table were investigated through measurement of the maximum shell displacement under direct force in different directions

#### Registration uncertainties MR/MR

The registration accuracy with mutual information algorithms has been discussed above in the section about uncertainty in target definition. Based on the high soft tissue contrast in the MR images and the similar information content in the reference and positioning image it was assumed that the accuracy is limited by the size of the voxels. A voxel size of 1.0 × 1.0 × 2.5 mm^3 ^gives a registration uncertainty of 0.5, 0.5, and 1.25 mm in the RL, AP and HF directions respectively.

## Results

### Uncertainties associated with MR transport

#### Calibration vector

The calibration vector relates the coordinate system in the MR scanner with the treatment table coordinate system. The estimated uncertainty for the calibration vector, based on the 16 independent measurements, was 0.5 mm, 0.4 mm resp. 0.8 mm in the RL, HF and AP directions. The mean value of the 16 observations is connected to a systematic uncertainty of 0.1 to 0.2 mm.

#### Correction for weight

The calibration vector was measured without load. Therefore there is a need to correct for the sagging of the treatment table under the weight of the patient. We found that the sagging of the treatment table could be modelled as a linear function of the patient weight (*w*) and the longitudinal coordinate for the prostate (*l*) in the MR coordinate system, according to:(2)

where the units are kg and mm respectively.

**Figure 4:**
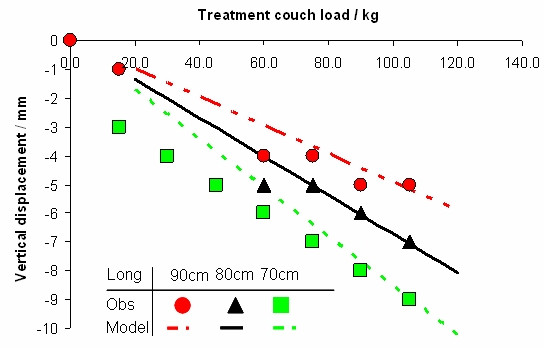
**Sagging of treatment table**. Modelled table sagging, the lines, is compared with observed sag, the points, for different simulated patient weights and prostate positions. The parameter "Long" describes the distance from the head end of the Miyabi shell to the prostate.

For simulated patients in the weight interval between 60 and 110 kg with their prostate located approximately 700-900 mm from the top of the skull, residual errors of maximum 1.2 mm was observed in the AP direction (figure 4), and 0.4 mm in the HF direction. In general the residual errors were small and the standard deviation of this systematic uncertainty was estimated to 0.6 mm in the AP direction, 0.2 mm in the HF direction, and neglectable in the RL direction.

#### Position reproducibility

Under direct force it was possible to displace the Miyabi shell slightly below 1 mm in the HF direction; this maximum displacement corresponds to an uncertainty under normal distribution assumption of around 0.5 mm. It was not possible to measure any positioning inaccuracies in the RL and AP directions. The uncertainty in the HF direction results in systematic uncertainties in the imaging for the treatment planning with a magnitude of 0.5 mm, and does in addition result in fraction to fraction positioning uncertainties of 0.7 mm (both MR and treatment table docking).

### Comparison with established technique

Table 1 summarizes results from the literature review in section 3 and results presented in section 4. The total estimated positioning uncertainty for a CT-based workflow, illustrated in figure 1a, is substantially larger than the estimated uncertainty using the MR-based workflow (figure 1b). The clinical implication of spatial uncertainties is the use of margins, dependent on both the random and systematic part. In the present work we use the model described through equation (1). The CT-based workflow should according to equation (1) be associated with the following margins: RL - 8.1 mm, AP - 8.7 mm, and HF - 10.7 mm. The corresponding margin for the MR-based workflow should be: RL - 5.3 mm, AP - 6.1 mm, and HF - 8.7 mm.

**Table 1:** Estimated positioning uncertainties CT resp. MR based treatment procedure

	CT based workflow						MR based workflow					
	CT/MR-systematic			CT/MR-Random			MR-systematic			MR-random		
Contributing factor	ΣRL mm	ΣAP mm	ΣHF mm	σRL Mm	σAP mm	σgHF mm	ΣRL mm	ΣAP mm	ΣHF mm	σRL mm	σAP mm	σHF mm
Prostate delineation	1.8	1.8	2.8	-----	-----	-----	1.8	1.8	2.8	-----	-----	-----
Geometrical distortions	0.2	0.2	0.2	-----	-----	-----	0.2	0.2	0.2	-----	-----	-----
MR to CT registration	2	2	2	-----	-----	-----	-----	-----	-----	-----	-----	-----
***Total treatment planning uncertainty***	***2.7***	***2.7***	***3.4***	**-----**	**-----**	**-----**	***1.8***	***1.8***	***2.8***	**-----**	**-----**	**-----**
Intra-fraction motion	-----	-----	-----	0.4	1.4	1.4	-----	-----	-----	0.4	1.4	1.4
CT to X-ray registration	-----	-----	-----	0.7	0.7	1.4	-----	-----	-----	-----	-----	-----
Fidutial marker uncertainty	1.0	1.0	1.0	-----	-----	-----	-----	-----	-----	-----	-----	-----
X-ray Imaging uncertainty	1.0	1.0	1.0	-----	-----	-----	-----	-----	-----	-----	-----	-----
MR Imaging uncertainty/distortion	-----	-----	-----	-----	-----	-----	0.5	0.5	0.5	0.5	0.5	0.5
MR to MR registration	-----	-----	-----	-----	-----	-----	-----	-----	-----	0.5	0.5	1.25
Calibration vector determination	-----	-----	-----	-----	-----	-----	0.1	0.2	0.1	-----	-----	-----
Weight correction	-----	-----	-----	-----	-----	-----	-----	0.6	0.2	-----	-----	-----
Docking mechanism	-----	-----	-----	-----	-----	-----	-----	-----	0.5	-----	-----	0.7
***Total Set-up uncertainty***	***1.4***	***1.4***	***1.4***	***0.8***	***1.6***	***2.0***	***0.5***	***0.8***	***0.7***	***0.8***	***1.6***	***2.1***

**Total uncertainty**	**3.0**	**3.0**	**3.7**	**0.8**	**1.6**	**2.0**	**1.9**	**2.0**	**2.9**	**0.8**	**1.6**	**2.1**

## Discussion

Through this literature review together with our analysis of the positioning procedure with MR, we claim that the MR-only treatment workflow, shown in figure 1b, allows for significantly smaller PTV margins than the CT-based workflow (figure 1a). This conclusion has been reached through estimations of the uncertainty for each sub process in the treatment chain and sum-up's of the total spatial uncertainty assuming that the errors from the sub processes are uncorrelated. This method yields results comparable to other studies, for example, the resulting margins for the positioning using CT-based workflow and gold markers are comparable with the results presented by Beltran et al. [42]. Excluding the uncertainty in the delineation of the prostate both Beltran et al. and the present study estimate the proper margins to between 4 mm and 5 mm in all directions. The contributions from different sources of uncertainty do however differ.

The reduced uncertainty does not necessarily mean that MR-only is the optimal workflow as other aspects also needs to be considered. It is not feasible to introduce a positioning method which requires considerably more patient time for all the 30-40 fractions than what are standard at many departments. However, the importance of occupation time per treatment would be reduced if the hypo-fractionation of prostate treatments becomes clinical standard.

The delineation uncertainty is dominating the systematic overall uncertainty also for the MR only workflow. It is clear that more effort needs to be spent on reducing uncertainty in the target delineation procedure.

In the present study we have used a generic algorithm for 3D distortions correction provided as a standard routine in the VB15 package delivered by Siemens. The accuracy of this correction was validated using a Philips PIQT phantom, through comparison with CT and through direct distance measurements in the images. The results were in agreement with the results reported by Krager et al. [23]. It can be expected that the accuracy of generic distortion correction algorithms may vary between individual scanners, it is thus important to validate the geometrical accuracy for each MR-scanner before any clinical implementation. Equally important is verification of the site specific registration accuracy, which can differ depending of algorithm, region of interest, and clinical implementation. The uncertainty quantification presented in Table 1 are representative for the described methodology, but should be verified locally.

Registrations between MR and CT, and MR to MR, were in the present study performed using a MI based method. An alternative workflow uses the internal gold markers as reference points in a landmark based registration. This registration method was not included in the present study for several reasons. -The markers are not clearly visible with the T2 weighted 3D sequence that is we use for target delineation. -Introduction of a dedicated sequence for visualization of the markers gives a systematic spatial uncertainty because of prostate movement between the sequences. -Use of a multi-echo sequence to acquire both T2 weighted images for delineation and proton density weighted images for visualization of the makers compromise the quality of the images used for delineation compared to present 3D sequence. -Finally, there is still a need for an in-depth investigation of the spatial uncertainties in the apparent marker position in the MR images, specifically, with respect to variations in frequency encoding direction, bandwidth, slice encoding method, and marker shape and orientation relative the main magnetic field.

## Conclusion

It was shown that, from a spatial uncertainty point of view, the MR-only prostate treatment workflow is to be preferred in front of a MR/CT-based procedure. The systematic uncertainties introduced by the MR/CT-registration are affecting the entire treatment but are avoided with the MR-based workflow, while the random uncertainties from fraction to fraction are approximately the same as for the MR/CT workflow.

## Competing interests

The authors declare that they have no competing interests.

## Authors' contributions

TN Participated in the design of the study participated in the literature review and drafted the manuscript. MN Participated in the design of the study and performed the experimental work

MGK Participated in the design of the study and in the literature review. MK Participated in the design of the study and in the literature review. All authors read and approved the final manuscript
